# Dramatic Changes in Thermoelectric Power of Germanium under Pressure: Printing *n–p* Junctions by Applied Stress

**DOI:** 10.1038/srep44220

**Published:** 2017-03-14

**Authors:** Igor V. Korobeinikov, Natalia V. Morozova, Vladimir V. Shchennikov, Sergey V. Ovsyannikov

**Affiliations:** 1 M. N. Miheev Institute of Metal Physics, Russian Academy of Sciences, Urals Division, 18 S. Kovalevskaya Str., Yekaterinburg 620137, Russia; 2 Bayerisches Geoinstitut, Universität Bayreuth, Universitätsstrasse 30, Bayreuth D-95447, Germany; 3 Institute for Solid State Chemistry, Russian Academy of Sciences, Urals Division, 91 Pervomayskaya Str., Yekaterinburg 620990, Russia

## Abstract

Controlled tuning the electrical, optical, magnetic, mechanical and other characteristics of the leading semiconducting materials is one of the primary technological challenges. Here, we demonstrate that the electronic transport properties of conventional single-crystalline wafers of germanium may be dramatically tuned by application of moderate pressures. We investigated the thermoelectric power (Seebeck coefficient) of *p**–*** and *n**–***type germanium under high pressure to 20 GPa. We established that an applied pressure of several GPa drastically shifts the electrical conduction to *p**–***type. The *p**–***type conduction is conserved across the semiconductor-metal phase transition at near 10 GPa. Upon pressure releasing, germanium transformed to a metastable *st12* phase (Ge-III) with *n**–***type semiconducting conductivity. We proposed that the unusual electronic properties of germanium in the original cubic-diamond-structured phase could result from a splitting of the “heavy” and “light” holes bands, and a related charge transfer between them. We suggested new innovative applications of germanium, e.g., in technologies of printing of *n–p* and *n–p–n* junctions by applied stress. Thus, our work has uncovered a new face of germanium as a ‘smart’ material.

Creation of new functional materials that are characterized by various combinations of physical and chemical properties, is one of the main research directions in materials science and engineering. Meanwhile, the well-known and commonly used materials can also uncover novel features which can also provoke emergent innovative applications, and for this reason, all the key materials are always in the focus of investigations. Tuning the electronic, optical, magnetic, and other parameters of materials by applied stress seems to be an effective strategy, which can lead to useful practical appliances. For instance, we can mention a recent progress in prediction and experimental realization of new metastable polymorphs with diverse opto-electronic characteristics in such key industrial semiconductors, as silicon^1^,^2^,^3^,^4^,^5^,^6^,^7^,^8^,^9^,^10^,^11^,^12^,^13^ and germanium 14,15,16,17,18.

A gradual turn from microelectronics to nanoelectronics and related miniaturization of constitutive elements of electronic devices designate new challenges and demand alternative methods in modification and control of properties. Although, moderate variations in temperature can lead to remarkable effects, e.g., to abrupt and reversible *p*–*n* inversion of electrical conduction type observed in Ag_10_Te_4_Br_3_ semiconductor at near 375 K^19^, more elegant external stimuli, such as controlled stresses would better suit the requests of micro- and nanoelectronics. Recent investigations demonstrated a certain progress in this area. For example, among others, it was reported that varying a stress value in thin films of InN one can tune its band gap^20^, or one can “write” electrically conducting zones on surface of silicon using a nanoindenter load^21^. Furthermore, surface indentation was proposed to be an effective strategy for mechanical recording of data^22^, and consequently, this approach was realized in IBM memory devices of ultrahigh storage density^23^.

A capability of control the electrical conduction by applied stress, and, in particular, a possibility of elegant switching between the different conduction types (*p*–, *n*–) or regimes, could substantially amplify the functionality of the existing semiconductor materials and their-based devises. On the other hand, it could stimulate emergent appliances. For instance, it has been established that conventional silicon, slightly doped with Ge (of ~1–3 at.%) acquires the pronounced properties of a ‘smart’ material and enables a simple and elegant *p–n* switching of its electrical conduction type by applied stress up to 3 GPa^24^. A recently discovered perovskite-type *ζ*-Mn_2_O_3_ semiconductor with a direct band gap of 0.45 eV also demonstrated a possibility of *p–n* switching under applied high pressure above 10–15 GPa^25^. Whereas, undoped silicon and other semiconductors for optoelectronics, such as GaAs, ZnSe and ZnTe did not reveal such effects^26^,^27^,^28^,^29^.

In this work we investigated the thermoelectric power of conventional single-crystalline germanium under applied high pressure to 20 GPa, that is, across the known semiconductor-metal phase transition from the cubic-diamond lattice (Ge-I) to a body-centred tetragonal *β*-Sn lattice (Ge-II) at near 10 GPa^30^,^31^,^32^,^33^,^34^,^35^,^36^,^37^,^38^,^39^,^40^,^41^,^42^,^43^,^44^,^45^,^46^,^47^,^48^,^49^. We have observed that a moderate applied pressure of several GPa dramatically shifts the electrical conduction to *p–*type. This effect may be well explained by a splitting of the “heavy” and “light” holes bands, and a related charge transfer between them. Whereas, samples recovered from higher pressure above 15 GPa, adopted a metastable simple tetragonal *st12* structure (Ge-III) with a semiconducting conductivity of *n–*type. These findings suggest that germanium has a strong potential for appliances in stress-related technologies, and one of the simplest examples of those, is ‘printing’ of the *n–p* diodes and *n–p–n* junctions on germanium surface using a variable applied stress.

## Results

**Figure 1:**
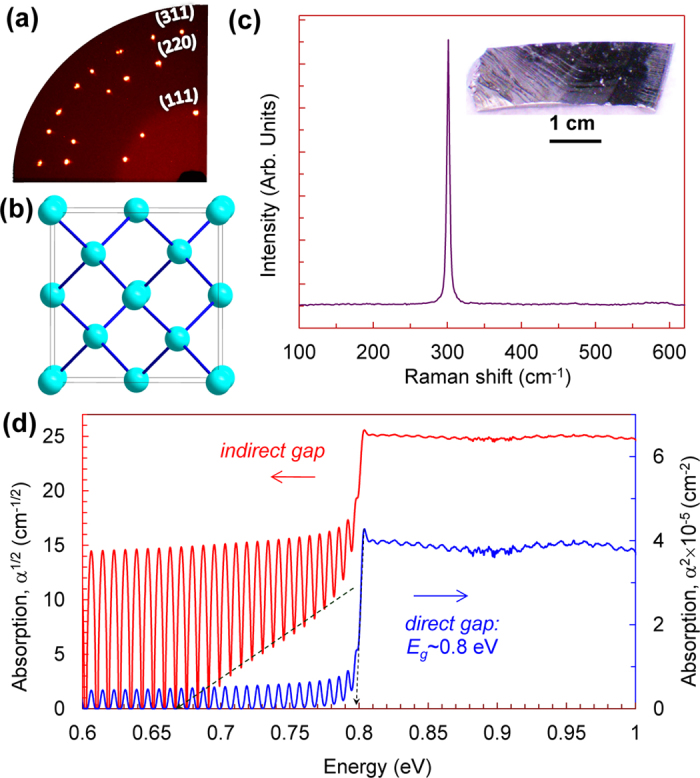
Structural and optical properties of single-crystalline samples of germanium at ambient conditions. (**a**) A quarter of X-Ray diffraction image of one of the samples and its indexing in the cubic diamond lattice. (**b**) The cubic diamond lattice of germanium (Ge-I). (**c**) Raman spectrum and photograph of one of the samples. (**d**) Near-infrared absorption (*α*) spectrum of one of the samples in two representations, as *α*^1/2^ and *α*^2^ for determination of indirect and direct band gaps, respectively.

A representative set of structural and optical data collected from the single-crystalline ingots of germanium is given in Fig. 1. All of these ingots crystallized in the diamond-type lattice (space group #227 –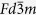) (Fig. 1a,b) and showed characteristic Raman peak at ~300 cm^−1^ (Fig. 1c). The near-infrared absorption spectra demonstrated a very abrupt absorption edge at near 0.8 eV (Fig. 1d). Using a standard expression for absorption edges in semiconductors with nearly parabolic energy bands, as follows^25^: 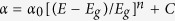 (where *α* is the absorption coefficient, *α*_*0*_ is a constant, *E* is the energy, *C* is an instrumental shift, and *n* ∼ ^1^/_2_ and ∼ 2 for direct and indirect band gaps, respectively), we established the direct band gap as of 0.8 eV (Fig. 1d). This value perfectly agrees with the literature data that address this gap to a direct electronic transition between the top of the valence band and the bottom conduction band at *Γ* point of the Brillouin zone^50^,^51^,^52^,^53^,^54^. However, germanium is known to have an indirect fundamental band gap of *E*_*g*_ = 0.67 eV^50^,^51^,^52^,^53^,^54^. Indeed, our absorption spectra suggested the existence of the indirect band gap somewhere between 0.6 and 0.7 eV (Fig. 1d), but this absorption edge was masked by the very strong resonance effects in our double-side-polished samples.

**Figure 2:**
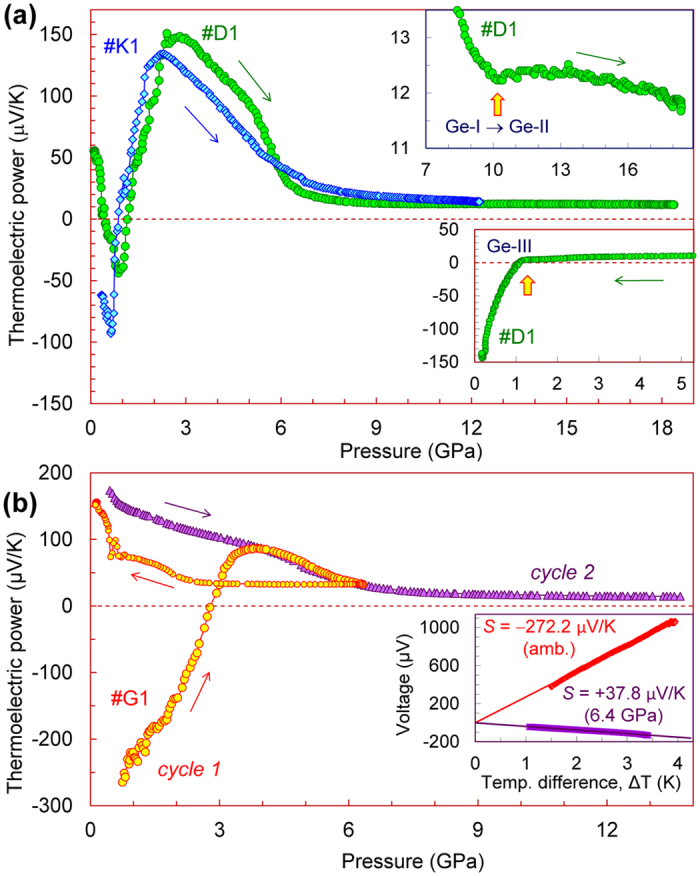
Pressure dependencies of the thermoelectric power (Seebeck effect) of three samples of germanium (#*K1*, #*D1*, and #*G1*). These curves were measured at room temperature in the cell with flat anvils. The thin arrows indicate the directions of pressure variation. (**a**) The upper inset shows a magnified part of the pressurization curve for sample #*D1* with the phase transition to the metal *β*-Sn-type phase (Ge-II) marked by an arrow. The lower inset displays a decompression dependence of the thermopower for this sample #*D1* and marks its jump at below 1 GPa by an arrow. (**b**) The dependencies are given for two successive pressure cycles. The inset shows examples of determination of the thermopower values (*S*) for the first cycle from linear slopes of a thermoelectric voltage (*U*) on a temperature difference (Δ*T*) as *S* = −*U/*Δ*T*.

**Figure 3:**
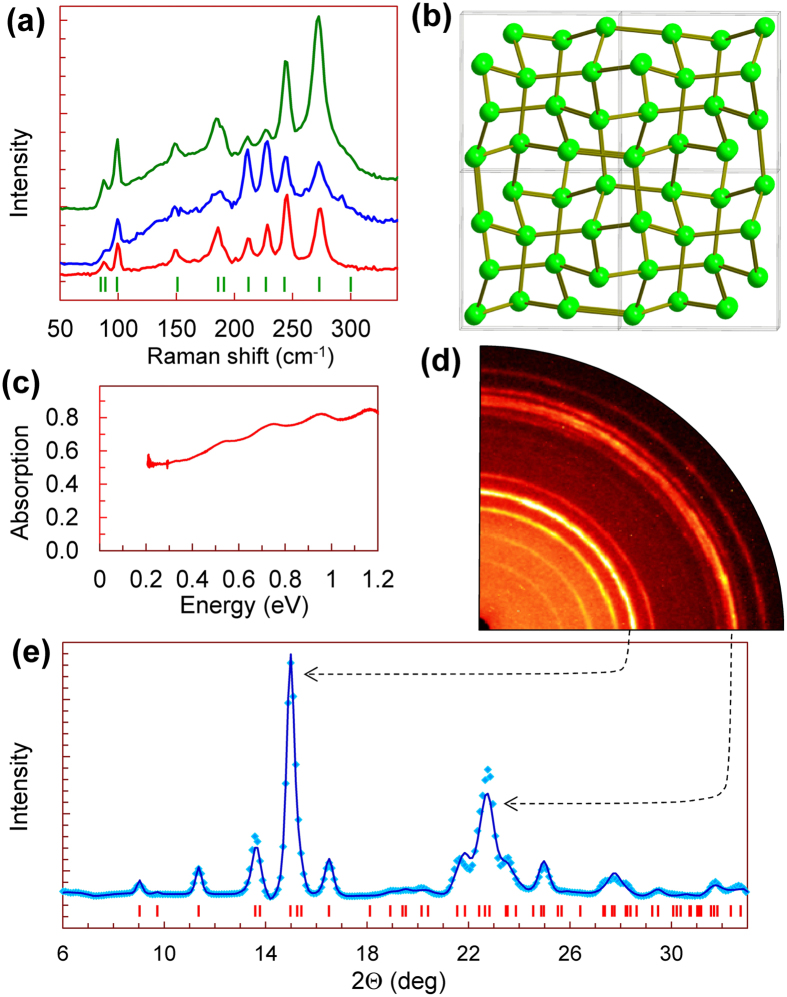
Structural and optical properties of the metastable simple tetragonal *st12* polymorph of germanium (Ge-III) recovered at ambient conditions. (**a**) Raman spectra collected from different areas of sample #*D1*. The dishes indicate the positions of Raman peaks for Ge-III taken from ref. 60. (**b**) Crystal structure of Ge-III projected down *c*-direction. (**c**) Absorption spectrum of sample #*D1* with *st12* structure. (**d**) A quarter of X-ray diffraction image collected from recovered sample #*D1*. (**e**) Rietveld refinement of X-ray diffraction pattern after background subtraction. The points are experimental data, the solid line is calculated profile, and the dashes are expected reflection positions for the *st12* structure of Ge-III. The residual parameter of this refinement was about *R*_*p*_ = 13.6%.

**Figure 4:**
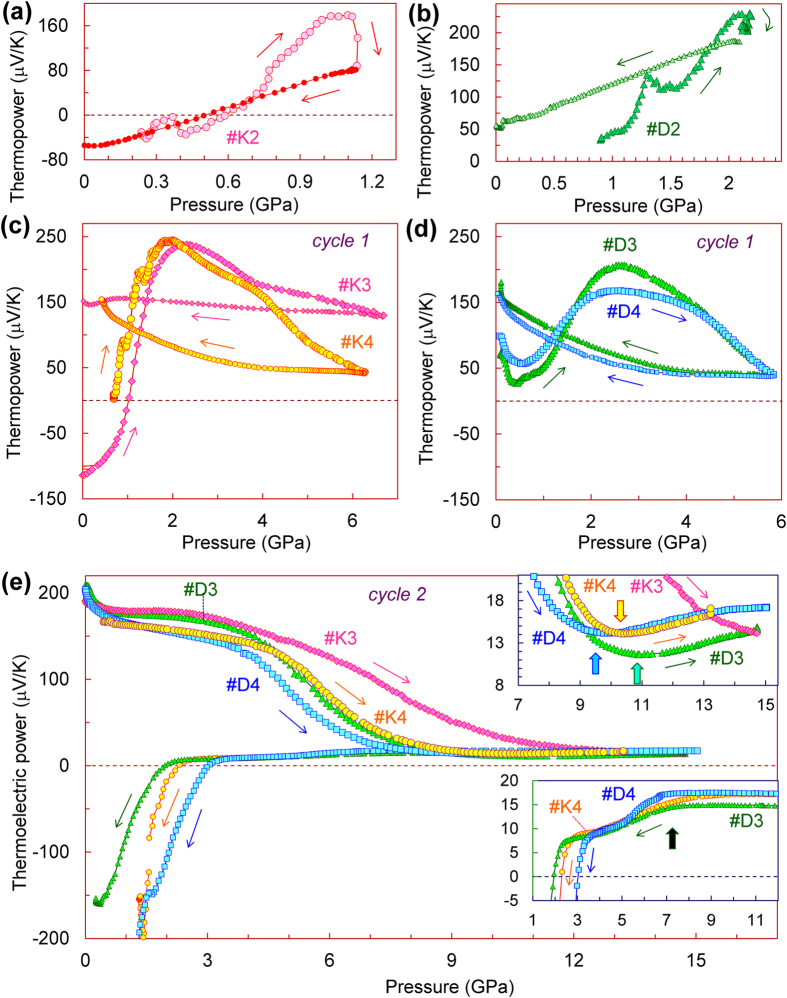
Pressure dependencies of the thermoelectric power (Seebeck effect) of the original germanium samples #*K2-#K4* and #*D2-#D4*. These curves were measured at room temperature in the cell with flat anvils. The thin arrows indicate the directions of pressure variation. (**a**) and (**b**) show that changes in the thermopower are reversible if applied pressures are less than 1–2 GPa. (**c**) and (**d**) show the irreversible changes in the thermopower for higher applied pressures up to 6–7 GPa. After the pressure was released the Seebeck effect turned to values as of +(150–200) μV/K. (**e**) The second pressure cycles across the phase transition to the metal *β*-Sn-type phase (Ge-II) (pointed in the upper inset by arrows). On decompression cycles germanium transformed to the metastable *st12* polymorph (Ge-III). The lower inset shows magnified parts of decompression curves, which exhibit distinct features at near 2–3 and 6–7 GPa.

**Figure 5:**
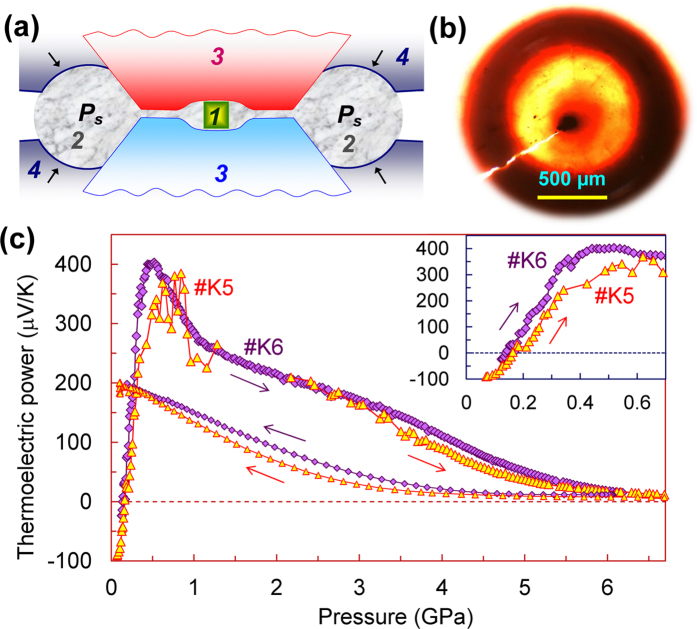
Pressure dependencies of the thermoelectric power (Seebeck effect) of the original germanium samples #*K5-#K6*. These curves were measured at room temperature in the cell with semispherical cavities in the anvils. (**a**) Side view of the cell with semispherical cavities in the anvils (***1*** – sample, ***2*** – sample container, ***3*** – anvils; ***4*** – supporting hard-alloy plungers, *p*_*s*_ means supporting pressure). (**b**) Photograph of a sample container with a sample (black piece) recovered after the high-pressure experiments. (**c**) Pressure dependencies of the thermopower for two samples. The thin arrows indicate the directions of pressure variation. The noise in thermoelectric signal from sample *#K5* around 1–2 GPa was likely related to issues with electrical probes. The inset shows magnified parts of these curves below 0.7 GPa.

**Figure 6:**
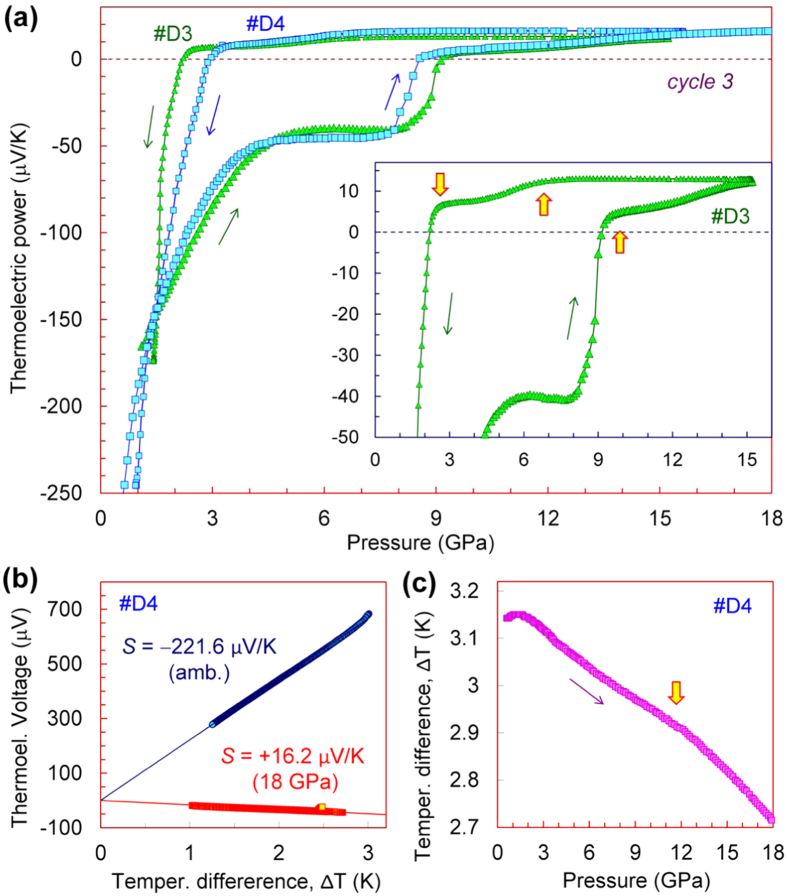
Pressure dependencies of the thermoelectric power (Seebeck effect) of the metastable *st12* polymorph of germanium (Ge-III). These curves were measured at room temperature in the cell with flat anvils. (**a**) These thermopower curves correspond to the third pressure cycles for samples #*D3* and *#D4* (the first and second pressure cycles are shown in Fig. 4d,e). The thin arrows indicate the directions of pressure variation. The inset shows a magnified part of the curves with the kinks pointed by the arrows. (**b**) Examples of determination of the Seebeck coefficient for sample *#D4*. (**c**) Pressure variation in temperature difference, *ΔT* along sample thickness; this curve corresponds to the thermopower dependence in (**a**) for sample *#D4*. The arrow marks the transition to the metal *β*-Sn-type phase.

We cut tiny chips from different ingots of germanium, labelled for convenience by *D*, *G*, and *K* letters (e.g., *#D1* – *#D4* are samples cut from ingot *D*) and measured their Seebeck coefficients as functions of applied high pressure up to 19 GPa (Fig. 2). This pressure range covered the known phase transition from the original cubic-diamond-type lattice (Ge-I) to the metal phase with the *β*-Sn-type lattice (Ge-II) at about 10 GPa^30^,^31^,^32^,^33^,^34^,^35^,^36^,^37^,^38^,^39^,^40^,^41^,^42^,^43^,^44^,^45^,^46^,^47^,^48^,^49^. As reported earlier, upon decompression, germanium can transform to one of its metastable polymorphs, instead of turning back to the cubic-diamond-type phase (Ge-I)^55^,^56^,^57^,^58^,^59^,^60^,^61^,^62^,^63^,^64^,^65^,^66^. To verify the crystal lattice of the recovered from high pressure samples, we examined them by Raman and X-Ray diffraction studies (Fig. 3). With pressure application, the thermopower of germanium samples demonstrated dramatic changes in its magnitude and sign in the stability region of the original cubic-diamond-type phase (Fig. 2). To explore these features in detail, we repeated the measurements for several more samples up to 7 GPa in two high-pressure cells with flat anvils and with toroidal anvils (Figs 4,5). In addition, we measured the thermopower of the metastable polymorph of germanium under high pressure to 18 GPa (Fig. 6). Below, we describe our findings in more detail.

### Thermopower of germanium in the semiconductor and metal phases

At ambient pressure the two ingots of germanium, labelled as *D* and *K*, exhibited a compensated electrical conduction with comparable hole and electron contributions. Meanwhile, the *D* ingot showed a slight preference to *p*-type, likewise, the *K* ingot – to *n*-type (Fig. 2a). The third ingot, labelled as *G*, was characterized by more pronounced *n*-type conduction at ambient pressure (Fig. 2b). Notice here, that germanium with one dominant type of charge carries typically has the larger Seebeck coefficients of about several hundreds of μV/K^67^,^68^. With pressure increase, the Seebeck coefficients of samples *#K1* and *#G1* demonstrated the *n–p* sign inversion at near 1 and 3 GPa, respectively (Fig. 2). Whereas, the sample *#D1* displayed even a double *p–n–p* sign inversion at the beginning of compression to 2 GPa (Fig. 2a). In general, under applied pressure all three samples demonstrated the similar maxima of their Seebeck coefficients at near 3–4 GPa, followed by a progressive drop of the thermopower value (Fig. 2). In the thermopower curve of sample *#D1* we detected a distinct kink at near 10 GPa, which may be attributed to the transition to the metallic *β*-Sn phase (upper inset in Fig. 2a). On the semiconductor-metal phase transition in silicon at a similar pressure value of 10 GPa, the thermopower exhibited the same feature^26^. In this metal *β*-Sn phase the Seebeck coefficient of germanium was weakly varied about *S *≈ +12 μV/K (Fig. 2a). Upon the decompression cycle, the thermoelectric power in sample *#D1* inverted its sign at near 1 GPa and tended to high negative values, suggesting a transition to a semiconducting phase (lower inset in Fig. 2a). On the contrary, the Seebeck coefficient of sample *#G1*, which was decompressed from 6 GPa, that is below the phase transition point of 10 GPa^30^,^31^,^32^,^33^,^34^,^35^,^36^,^37^,^38^,^39^,^40^,^41^,^42^,^43^,^44^,^45^,^46^,^47^,^48^,^49^, kept positive values and turned to *S* ∼ +150 μV/K after the pressure was released (Fig. 2b). On the re-pressurization cycle the sample *#G1* behaved already as a *p*-type material (Fig. 2b).

### Metastable phases of germanium

To determine the crystal structure of the recovered from high pressure samples we examined them by Raman spectroscopy and X-ray diffraction. In Fig. 3 we display these data on example of recovered sample *#D1* which turned to a slightly textured polycrystal. The Raman spectra collected from different points at its surface exhibited peaks at 88, 99, 149, 185, 191, 212, 228, 244, 273, and 300 cm^−1^ (Fig. 3a). The intensities of these Raman peaks were strongly varied from point to point (Fig. 3a), thereby indicating that the spectra are highly sensitive to orientation of the crystal grains. These Raman spectra well agreed with those observed in previous works for a metastable polymorph of germanium, prepared either in diamond anvil cells^60^, or by a surface nanoindentation^61^,^62^. In the literature, these spectra were attributed to a simple tetragonal lattice with 12 atoms per unit cell (*st12*, space group #96 – *P*4_3_2_1_2) (also known as Ge-III)^60^,^61^,^62^. Whereas, other papers reported different Raman spectra, e.g., a strong peak at near 200 cm^−1^, and addressed them to another metastable polymorph with a body-centred cubic lattice with 8 atoms per unit cell (*bc8*, Ge-IV)^63^,^64^,^65^,^66^. Earlier investigations noticed that the formation of the metastable polymorphs in germanium is controlled by both a decompression rate^58^ and stress conditions^57^. The Rietveld refinement of the X-ray diffraction pattern collected from the recovered from high pressure sample *#D1* confirmed the tetragonal *P*4_3_2_1_2 structure of Ge-III (Fig. 3d,e). We found its unit cell parameters and atomic coordinates as follows: *a* = 5.927(2)Å, *c* = 6.969(6)Å, *V* = 244.8(5)Å, *Z* = 12, Ge1[0.093(3),0.093(3),0], and Ge2[0.170(8),0.363(6),0.255(6)] (Fig. 3b). These parameters were similar to those reported earlier for this phase^39^. Remarkably, that besides this tetragonal Ge-III phase, the recovered sample *#D1* exhibited no traces of any other phases. The other two samples, *#G1* and *#K1*, after their recovery from high pressures were mixtures of both the original and the tetragonal *st12* phases. These facts show that for preparation of a pure *st12* phase one should apply a high pressure well above the phase transition point of 10 GPa^30^,^31^,^32^,^33^,^34^,^35^,^36^,^37^,^38^,^39^,^40^,^41^,^42^,^43^,^44^,^45^,^46^,^47^,^48^,^49^.

The thermopower curve for sample *#D1* demonstrated that upon pressure releasing at below 1 GPa it turned to a semiconductor with the dominant *n*–type conduction (lower inset in Fig. 2a). Earlier band structure calculations predicted that the *st12* phase of germanium should be a direct-band-gap semiconductor with an energy gap of *E*_*g*_ = 0.7 eV^59^. The major part of recovered sample *#D1* crystallizing in this *st12* structure (Ge-III) presented a thin disc-shaped film of ~15 μm in the thickness and of ~150 μm in the diameter, and, hence, we could proceed with examination of its electronic band structure by means of near-infrared absorption spectroscopy (Fig. 3c). However, these spectra did not reveal any absorption edges. But it should be noted here that numerous defects and high concentrations of free carriers associated with these defects often impede direct observation of absorption edges in polycrystalline semiconductors. To obtain the metastable polymorph of germanium in the larger amount for more detailed investigations, we tried to prepare that by means of a high-pressure high-temperature synthesis at pressure near 20 GPa in large-volume presses. The bulk samples recovered from these syntheses were apparently multi-phased and seemed to be more appropriate for investigations of a “zoo” of germanium polymorphs. Hence, detailed investigations of the metastable *st12* phase were left beyond the scope of the present work.

### Tuning the thermopower in germanium

To examine the discovered pressure-driven thermopower inversion in detail, we measured pressure dependencies of the thermopower for several more samples of germanium (Fig. 4). We can summarize our findings and conclusions, as follows:

1. Tuning the thermopower by applied pressure up to 1 GPa seems to be reversible (Fig. 4a). It was seen, for instance, in sample *#K2*, in which we observed an *n*–*p* inversion at near 0.55 GPa under compression to 1.1 GPa and then upon pressure releasing (Fig. 4a). Sample *#D2* decompressed from about 2.1 GPa showed a certain positive shift in the thermopower value after the pressure was released (Fig. 4b). In another sample recovered from a higher pressure of 3 GPa, this positive shift became more sizable and the Seebeck coefficient turned to a value of *S* ∼ +100 μV/K (not shown).
2. Treatment by the higher pressures, ranging between 4 and 9 GPa, that is, above the flat extremum in the thermopower curves, always results in the irreversible turn to the *p–*type conduction (Figs 2b and 4c,d). After the pressure is released, the Seebeck coefficient comes to values of *S* ∼ +(150–200) μV/K.
3. High-pressure treatment somewhat above the semiconductor-metal phase transition point (10 GPa)^30^,^31^,^32^,^33^,^34^,^35^,^36^,^37^,^38^,^39^,^40^,^41^,^42^,^43^,^44^,^45^,^46^,^47^,^48^,^49^, leads to the formation of the metastable *st12* polymorph (Ge-III), which is characterized by the dominant *n–*type conduction and by the Seebeck coefficient as of *S* ∼ −(150–250) μV/K (Fig. 4e, lower inset in Fig. 2a).

Although, we found a very good consistency in the thermopower data collected for different germanium samples, the extrema around 2–4 GPa were noticeably varied from sample to sample (Figs 2,4). The thermopower curve for sample *#K3* above 4 GPa showed a sizable deviation from the curve for sample *#K4* (Fig. 4c), thereby indicating that the appearance of pressure gradients leads to smearing of the thermopower extremum. The appearance of these pressure gradients is related to a strong shifting of the sample from the central area of the limestone container (Fig. 5b) to one of its edges.

For comparison we measured the Seebeck coefficients of two more samples, *#K5* and *#K5* under pressure up to 6–7 GPa in the high-pressure cell with concave anvils, which provided more uniform quasi-hydrostatic pressure conditions (Fig. 5a,b). The pressure dependencies of the thermopower measured in this cell also displayed the *n*–*p* sign inversion followed by the pronounced extremum (Fig. 5c). But compared to the data gathered in the cell with flat anvils from the samples cut from the same ingot *K (#K1, #K3, #K4*) (Figs 2a and 4c), these features in the thermopower behaviour were apparently shifted to the lower pressures (Fig. 5c). Thus, the Seebeck coefficient of germanium, measured in the cell with concave anvils demonstrated an anomalously high pressure derivative as of about 1000 μV/GPa from ambient pressure to 0.5 GPa (inset in Fig. 5c). After the pressure was released the originally *n*–type germanium also turned to *p*–type and demonstrated the Seebeck coefficient as of *S* ~ +200 μV/K (Fig. 5c). Comparing the data collected in the two different high-pressure cells, we can conclude that the presence of minor non-hydrostatic stresses can partly suppress and smear the extremum in the pressure dependence of the thermopower (Figs 2 and 4a,b).

### Thermopower of the metastable polymorph of germanium

As we have verified in this work, the compression of germanium in our cell to pressure values somewhat above the semiconductor-metal phase transition point (10 GPa)^30^,^31^,^32^,^33^,^34^,^35^,^36^,^37^,^38^,^39^,^40^,^41^,^42^,^43^,^44^,^45^,^46^,^47^,^48^,^49^ followed by gradual decompression, led to its transmutation into the metastable *st12* polymorph (Ge-III) (Fig. 3). After the decompression cycles for the second pressure runs we could *in-situ* prepare the metastable *st12* phase in samples *#D3*, *#D4* and *#K3* (Fig. 4e), and then, we performed the re-pressurization runs for this Ge-III polymorph (Fig. 6a). These dependencies for Ge-III polymorph demonstrated rather spectacular features, and at about 10 GPa they suggested a transition to the metal phase (Fig. 6a). This scenario looked resembling to the semiconductor-metal phase transition in the original cubic-diamond-type phase (upper insets in Figs 2a and 4e). It was interesting to note that in some pressure dependencies of the temperature difference (Δ*T*) along the sample thickness, one could see the pronounced bends at near 10 GPa (Fig. 6c). Since, the Δ*T* value depends on sample thickness (*h*) and its thermal conductivity (*λ*) as: Δ*T* ~ *h*/*λ*, this bend in the Δ*T* curves might be linked to enhancement of the thermal conductivity in the metal *β*-Sn-type phase.

The thermopower curves for the metastable *st12* polymorph (Ge-III) apparently indicated the existence of an intermediate electronic (or structural) phase, which was observed between 4 and 8 GPa on pressurization, and between 7 and 2 GPa on decompression cycles (Fig. 6a). It is interesting to recall here, that in case of silicon, a return transition from a metal *β*-Sn phase to a semimetal rhombohedral *r8* phase (Si-XII)^60^,^69^,^70^,^71^, was well detectable in pressure dependencies of the thermopower, by a gradual lowering in its value at near 7–9 GPa upon decompression^26^. A feature we discovered in germanium at near 7 GPa upon pressure releasing (insets in Figs 6a and 4e) looked very similar to that in silicon^26^, although, the rhombohedral *r8* phase has not yet been observed in germanium. At about 2 GPa on pressure releasing, silicon transforms to a semimetal *bc8* phase (Si-III) with a *p*-type electrical conduction.^72^ Previous work showed that on this transition the positive Seebeck coefficient of silicon abruptly raises to magnitudes of *S* ∼ +(15–20) μV/K^26^. On the contrary, the thermopower of germanium, below ~2 GPa drastically changed its sign and turned to high negative values (Fig. 6a). We could verify by Raman spectroscopy that these samples *#D3* and *#D4* after the pressure was released, crystallized in the *st12* phase (Ge-III). It was reported in the literature that the *β*-Sn → *st12* phase transition in germanium under decompression begins already at about 7–9 GPa^57^,^60^. These pressure values have correspondence with the above-discussed minor lowering in the thermopower value we observed in germanium at below 7 GPa, but not with the thermopower jump at below 2 GPa (inset in Fig. 6a). Thus, the crystal structure of the intermediate phase we found in germanium at pressures between 4 and 8 GPa on pressurization and between 7 and 2 GPa on decompression cycles (Fig. 6a), cannot be figured out at the moment. Potentially, it could be the same tetragonal *st12* phase but with the dramatically modified electronic band structure and reduced or even closed energy band gap.

## Discussion

The Seebeck coefficient (*S*) of an intrinsic non-magnetic semiconductor linearly depends both on its band gap value (*E*_*g*_) and on the ratio of hole (*σ*_*p*_) and electron contributions (*σ*_*n*_) to electrical conduction, and in a simple two-band case, it comes as follows^73^:


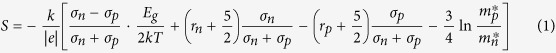


where, *k* is the Boltzmann’s constant, *e* is the electron charge (*k*/|*e*| ≈ 86.4 μV/K), *T* is the temperature, *r*_*n*_(*r*_*p*_) and 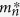
(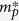) are the scattering parameters and the effective masses of density of states of electrons (holes), respectively. The samples of germanium we investigated are intrinsic semiconductors, and, hence, their behaviour can be analysed in the framework of this model. Both indirect and direct band gaps in the cubic-diamond-structured phase of germanium, were reported to widen with pressure, with the coefficients of about 4 meV/GPa for the indirect gap,^50^,^51^ and of 120 meV/GPa for the direct one^52^,^54^. As seen from Eq. 1 these moderate changes in the band gaps cannot explain the anomalous pressure dependencies of the thermopower (Figs 2,4, and 5). Hence, these thermopower inversions may be attributed only to variations in the *σ*_*p*_*/σ*_*n*_ and 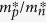 ratios (Eq. 1). For germanium samples with pure *n*–type conduction (i.e., *σ*_*p*_ = 0), and with the typical values of the scattering parameter as of *r*_*n*_−½ and *E*_*g*_ = 0.67 eV,^50^,^51^,^52^,^53^,^54^ the Seebeck coefficient should be larger than −1 mV/K. In sample *#G1* (Fig. 2b) the thermopower value at ambient conditions was only *S*∼−270 μV/K, thereby suggesting the *σ*_*p*_*/σ*_*n*_ ratio as of 0.63 (Eq. 1). The electrical conduction of the other two bulk samples, *#D* and *#K* of which Seebeck coefficients were of about ±50 μV/K at ambient pressure (Fig. 2a), was apparently compensated (*σ*_*p*_ ≈ *σ*_*n*_).

Thus, the pressure-driven shift to the *p*–type conduction in germanium (Figs 2,4,5) should be related to enhancement of the hole partial contribution (Eq. 1). This hole contribution to the conduction is determined by *σ*_*p*_ = *μ*_*p*_*p*, where *p* is the “effective” concentration of hole carriers and *μ*_*p*_ is their “effective” mobility value^73^. As the fundamental band gap of germanium only slightly widens with pressure^50^,^51^, the concentration of charge carriers, which in intrinsic semiconductors, are linked to native point defects in crystal lattice, unavoidable impurities, and those carriers which are thermally-activated over a band gap, should not increase with pressure. Therefore, the high positive values of the Seebeck coefficient near 1–4 GPa (Figs. 2, 4, and 5) indicating that the *p*–type conduction becomes dominating, may be related to increase in hole mobility values.

As stated in the literature, the top of the valence band of germanium at *Γ* point of the Brillouin zone consists of two overlapping hole bands of so-called “light” and “heavy” holes with typical effective masses of about 0.043*m*_*0*_ and 0.33*m*_*0*_, respectively. Several previous works claimed experimental observations of distinct crossovers in the electronic band structure of germanium under applied pressure of 2–3 GPa^74^,^75^,^76^,^77^. For instance, it was found that the electrical conduction of *n*–Ge is moderately diminished with pressure to 2 GPa^74^ or 3 GPa^75^, in agreement with the minor widening in its band gap value 50,51, but above this pressure point the electrical conduction begins to increase with pressure^74^,^75^. Another work discovered kinks at 1.8 GPa in pressure dependencies of phonon energies of germanium and addressed them to band structure reconstruction^76^. The last paper speculated that with pressure application the bottom of the *Δ* valley of the conduction band of germanium shifts below the bottoms of the *Γ* and *L* valleys, and hence, its fundamental indirect band gap becomes related to the transition between the bottom of this *Δ* valley and the top of the valence band at the *Γ* point of the Brillouin zone^76^. Dramatic changes in electronic transport properties of germanium found near 3 GPa in one more work, were also addressed to the intervalley transition^77^. Meanwhile, it should be also noted that some other studies of the electronic transport properties of germanium did not find any remarkable features across the above pressures^78^. One more paper, considering the anomalous behaviour of germanium in the cubic diamond phase, proposed a possibility of pressure-stimulated transfer of the hole carriers from the “heavy” holes band to the “light” one^79^,^80^. The mobility values of carriers of the “light” holes band should be essentially higher than those of carriers of the “heavy” holes band, and hence, upon this transfer the hole partial contribution to the electrical conduction should be significantly enhanced. In a line with this conjecture, two recent studies on “compressively strained” by Sn-doping germanium^81^ and strained films of pure germanium^82^ clearly documented the above proposed splitting of the “heavy” and “light” holes bands.

The abrupt pressure-driven *n*–*p* inversion and the high positive values of the Seebeck coefficient we observed at pressures of 1–5 GPa (Figs 2, 4, and 5) indicated a dramatic enhancement of the hole partial conduction (Eq. 1). This feature may be well explained by the above-discussed splitting of the two holes bands under applied pressure and a following transfer of the carriers from the “heavy” holes band to the “light” one. This model can also explain the anomalously high pressure derivative of the Seebeck coefficient we documented (Inset in Fig. 5c) as well as the crucial influence of minor non-hydrostatic stresses, which was seen from the comparison of the data obtained in the two different cells (Figs 2a, 4c,d, and 5a). One can surmise that this fine reconstruction of the band structure in germanium should be limited by available free hole carriers. Therefore, the pronounced pressure-driven *n–p* inversions we revealed in this work (Figs 2, 4, and 5), may be well visible in samples with *intrinsic* semiconductor conductivity. Whereas, in strongly doped samples of *n*–type, such a pressure-driven *n–p* sign inversion is unlikely to be observable, although, some anomalies in the properties resulting from the splitting of the holes bands still may occur.

The irreversible shift to the *p*-type conduction observed in the samples recovered from high pressures below the Ge-I → Ge-II phase transition point, i.e., in the cubic-diamond-type phase (Figs 2, 4c,d, and 5a), is most likely related to the conservation of residual strains which can keep a splitting of the “light” and “heavy” holes bands after the pressure is released. But it should be also noted, that applied high pressures could produce a number of “damages” in the crystal lattice, and hence, a native defect structure of the crystals might be moderately modified under pressure. Earlier studies of an impact of fast-neutron bombardment of germanium revealed that point damages in its crystal lattice lead to *p*–type conduction^83^,^84^. These results are in line with our findings (Figs 2, 4, and 5). Theoretical investigations of potential point defects which may be formed under external mechanical impacts on the crystal lattice of germanium, found two energetically favourable self-interstitial defects, such as: (*i*) a “split-interstitial” configuration which is electrically neutral, and (*ii*) an “open cage” configuration which has a donor-type^85^,^86^. Thus, we cannot infer which sorts of defects could potentially contribute to enhancing the *p*-type conduction, but their contribution could not be significant. The *n*–type conduction we established in the *st12* metastable polymorph (Ge-III) (Figs 2a, 4e, 6a) indicates that the native defects in its crystal structure are mainly of an electron type.

**Figure 7:**
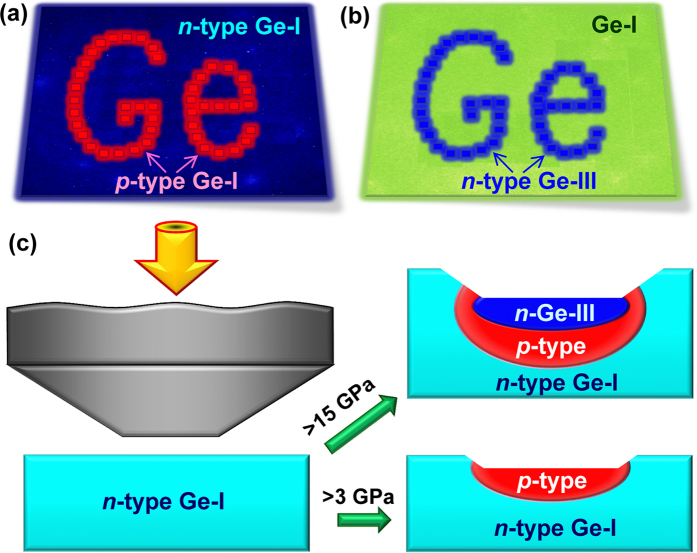
Potential applications of stress effects in germanium. (**a**) ‘Printing’ of *p**–***type zones on *n**–***type surface of conventional germanium. (**b**) ‘Printing’ of *n**–***type zones with the metastable *st12* structure (Ge-III) on surface of conventional germanium with compensated conduction (*σ*_*n*_ ≈ *σ*_*p*_). (**c**) Schematic view of hard tip for ‘printing’ and profile depths of ‘printed’ zones in dependence on applied stress value. Because of a stress distribution inside the material, such a ‘printing’ can form multilayered structures with alteration of different conduction types (e.g., *n**–**p**–**n*).

The dramatic changes in the thermopower of conventional germanium we revealed in this work, suggest novel possible innovative applications of this material. Among those, we can anticipate different micro- and nanoscale junctions with stress-controlled properties, embedded in various integrated circuits. A simple example of such junctions is a stress-controlled *n–p* switch. Using some designed printer-type device with a set of hard tips, one can “print out” circuits and zones of different conduction types on surface of germanium. The simplest examples of such ‘printing’ can be (*i*) a ‘writing’ of a thin *p–*type layer on a surface of *n–*type germanium (Fig. 7a), or (*ii*) a fabrication of a thin *n–*type layer of the metastable Ge-III polymorph on a surface of conventional germanium with the cubic-diamond-type structure (Fig. 7b). In the latter case, a stress distribution in the material should lead to the fabrication of an intermediate *p–*type layer of the cubic-diamond-type germanium between this *n–*type Ge-III layer and the substrate, as shown in Fig. 7c. Varying the geometrical parameters of the printing tips and conditions of load/unload, one can modify the profile depths of such multi-layered structures. Earlier investigations have already discovered that applied stress can remarkably tune the electronic properties of germanium. For instance, it was predicted that controlled tensions along < 111 > directions can turn germanium to a direct band gap semiconductor^87^; experimentally, this strategy was realized in thin films^88^.

Above 10 GPa in the metal *β*-Sn-type phase of germanium, the Seebeck coefficient in different samples varied between *S* ~ +5 and +17 μV/K (insets in Figs 2a, 4e and 6a). The lowest values were measured in the samples undergoing the transition to the metal Ge-II phase from the metastable Ge-III one, with a concurrent *n–p* sign inversion in the Seebeck effect (Fig. 6a). But upon the phase transition from the original cubic-diamond-type Ge-I phase with the *p*-type conduction, the thermopower values of the metal Ge-II phase were essentially higher, of *S* ~ +(11–17) μV/K (inset in Fig. 4e). This difference can be explained by the fact that upon the reconstructive transition to the *β*-Sn-type metal phase, the samples passed via a region of the phase coexistence, and hence, above 10 GPa the contributions of either the *p*-type Ge-I or the *n*-type Ge-III phases were still considerable. Meanwhile, all the samples demonstrated the similar uptrends in their pressure curves of the thermopower in the metal *β*-Sn phase (insets in Figs 4e and 6a). This behaviour should be related to a gradual band structure reconstruction. For “simple” metals with weakly changeable band structures, the volume contraction is expected to lead to a moderate decrease in the absolute value of the thermopower because of a proportional increase in the “effective” free carrier concentration^89^. However, even elemental metals deviate from this trend^90^, thereby indicating that band structure modifications make a major contribution to pressure dependencies of their Seebeck coefficients. Re-visiting the thermopower data for the metal *β*-Sn-type polymorph of germanium, we can conclude the following: (*i*) the Ge-I → Ge-II phase transition was best seen in sample #*D1* at 10 GPa (Fig. 2a), (*ii*) the thermopower value of the pure *β*-Sn-type phase is about *S* ≈ +12 μV/K, and (*iii*) after the phase transition is completed, the pressure behaviour of the thermopower of the *β*-Sn-type phase corresponds to the above predictions for “simple” metals (inset in Fig. 2a)^89^.

The high values of the Seebeck coefficient of the metastable Ge-III polymorph we found in the present work (Fig. 6a,b) suggest that this phase can be a narrow-band-gap semiconductor with a certain potential for the thermoelectricity. It should be noted here that Ge-rich materials, and in particular, Si_1−x_Ge_x_ alloys, are known to be excellent thermoelectrics^91^,^92^,^93^,^94^,^95^,^96^,^97^,^98^,^99^. We have verified that this metastable *st12* polymorph (Ge-III) in our thin samples recovered from high pressure persisted at least for several years. Probably, the local strains in the recovered samples help to retain this metastable high-pressure structure at ambient pressure.

## Conclusions

We have measured the Seebeck effect of single-crystalline samples of germanium with intrinsic electrical conduction of both *p**–*** and *n**–***types under high pressure to 20 GPa at room temperature. We have established that applied pressure strongly shifts the conduction to *p**–***type in the original semiconductor phase, and then, the *p**–***type conduction is further conserved in the metal *β*-Sn-type phase. Upon pressure releasing, the *β*-Sn-type phase transformed to the *st12* metastable polymorph (Ge-III) with the *n**–***type semiconducting conductivity. We have addressed the shift to the *p*–type conduction in the cubic-diamond phase to a pressure-driven splitting of the overlapped “heavy” and “light” holes bands, stimulating a charge transfer to the “light” band with more mobile hole carriers. In addition, we have verified that this *n**–**p* sign inversion is reversible if applied pressure is less than 2 GPa, and under higher applied pressures it becomes irreversible. Thus, our work has clearly demonstrated that the electronic transport properties of germanium may be dramatically tuned by a moderate applied stress. This finding can stimulate novel innovative applications of germanium as a ‘smart’ material. We have suggested that germanium may be utilized, for instance, in stress-controlled *n–p* switches and in technologies of ‘printing’ of *n–p* and *n–p–n* junctions by applied stress.

## Experimental section

For investigations we used several conventional bulk single-crystalline ingots of germanium from different suppliers. For convenience, we labelled these bulk samples by *D*, *G*, and *K* letters, and consequently numbered small microscopic samples cut from these ingots (e.g., samples cut from ingot *D* as *#D1* – *#D4*). The carrier concentrations in these bulk ingots were about 10^14^ cm^−3^. We also synthesized two bulk samples of germanium from conventional powder at 20 GPa and 600 °C using a 1200-tonne multi-anvil press at Bayerisches Geoinstitut. Both the original crystals and samples recovered after the high-pressure experiments were characterized by standard structural and optical techniques (Figs 1, 3). The crystal structure of the samples was verified in X-Ray diffraction studies performed on a high-brilliance Rigaku diffractometer (Mo Kα radiation) equipped with Osmic focusing X-ray optics and Bruker Apex CCD detector. In addition, we examined the crystal structure of the samples by Raman spectroscopy using two setups. In one of them the Raman spectra were excited with the 514.5 nm line of an Ar laser and analyzed by a Renishaw Ramascope; in another one the spectra were excited with the 632.8 nm line of a He-Ne laser and analysed by a Labam spectrometer. The electron structure of the samples was examined by near-infrared absorption spectroscopy using a Bruker IFS 120 Fourier transform spectrometer For the absorption studies the original samples were double-polished to the thickness of about 15–20 μm; the samples recovered after the high-pressure experiments had similar thicknesses.

The measurements of the thermopower (Seebeck coefficient) under high pressures (Figs 2, 4, 5, 6) were carried out on a fully automated high-pressure setup^100^. This setup presented a mini-press that smoothly generated an applied force to a high-pressure cell with a sample. Several nanovoltmeters and other sensor devices were connected to the cell for recording of all relevant parameters of a sample and environment^100^. This type of measurements enabled to follow the properties evolution under nearly continuous variation in pressure. A force applied to the high-pressure cell was automatically measured *in-situ* by a digital dynamometer directly on the cell. Then, a pressure value on a sample was automatically estimated from a calibration load curve based on the well-known and distinctly observable phase transitions^101^.

We utilized two different anvil-type high-pressure cells of the modified Bridgman-type^102^. In these cells a sample container made of the limestone (soft CaCO_3_-based material) served both as a pressure-transmitting medium and as a gasket to keep a sample in the space between the anvils^103^. A high and uniform pressure was generated in the central area of the sample container. In visual examinations of sample containers recovered after the high-pressure experiments we verified the sample position (Fig. 5b). The majority of the experiments were performed in a cell with flat anvils made of sintered diamonds with typical working diameters (culets) of ~600 μm^104^. We loaded in this cell a thin disc-shaped sample with typical sizes of about 200 × 200 × 30 μm^3^. In another cell the both anvils had a semispherical cavity in their central parts that enabled to provide a more uniform pressure in a larger volume (Fig. 5a)^103^,^105^. In the latter cell we loaded bulk samples with typical sizes of about 150 × 150 × 150 μm^3^. To produce a temperature difference (Δ*T*) of a few Kelvin along the sample thickness, the upper anvils in both cells, were heated up by electrical current heaters. This temperature difference was measured by means of thermocouples attached to the fixed points at the anvils. A relative uncertainty in determination of the Seebeck coefficient values by this method was related to a potential inaccuracy in estimation of the above mentioned Δ*T* value, and it was found to be less than 5%^106^. We monitored that the outcome thermoelectric signal was caused exclusively by the produced temperature difference, Δ*T* (Fig. 6b). Therefore, parasitic thermal and electrical signals did not make any noticeable contributions to the thermoelectric signal, and hence, the absolute error in determination of the thermopower should be less than 0.5 μV/K^106^. Other details of the high-pressure thermopower technique were similar to those described in recent previous works^106^,^107^.

## Additional Information

**How to cite this article**: Korobeinikov, I. V. *et al*. Dramatic Changes in Thermoelectric Power of Germanium under Pressure: Printing of *n–p* Junctions by Applied Stress. *Sci. Rep.* **7**, 44220; doi: 10.1038/srep44220 (2017).

**Publisher's note:** Springer Nature remains neutral with regard to jurisdictional claims in published maps and institutional affiliations.
